# The 100 Most Cited vs. Most Relevant Articles in the Journal of Neurosurgery: A Bibliometric Analysis

**DOI:** 10.7759/cureus.4498

**Published:** 2019-04-18

**Authors:** Bolin Liu, Shujuan Liu, Anthony JG Alastra, Deependra Mahato, Emilio C Tayag, Vladimir A Cortez, Javed Siddiqi

**Affiliations:** 1 Neurosurgery, Xi'an International Medical Center, Xi'an, CHN; 2 Obstetrics and Gynecology, Xijing Hospital, Xi'an, CHN; 3 Neurosurgery, Desert Regional Medical Center, Palm Springs, USA; 4 Neurology and Neurosurgery, Desert Regional Medical Center, Palm Springs, USA; 5 Neurosurgery, Redlands Community Hospital, Redlands, USA

**Keywords:** bibliometrics, citation analysis, neurosurgery, relevancy

## Abstract

Introduction

The Journal of Neurosurgery (JNS) published its first volume in 1944 and has evolved into the top cited journal in the field of neurosurgery. The aim of this study was to determine and characterize the 100 most cited (based on the total number of citations) vs. most relevant (based on the number of citations per year) articles originating in JNS.

Methods

The top 100 most cited articles in JNS were determined by searching the Web of Science database. Citations per year were additionally calculated for the top 1000 articles by total citations to rank the 100 most relevant articles.

Results

The median number of total citations for the 100 most cited articles in JNS was 505 (range 383-2200), and the median number of citations per year for the 100 most relevant articles was 21.88 (range 17.31-82.61). The median year of publication for the 100 most cited and most relevant articles was 1990 and 1999, respectively (P < 0.0001). Most articles originated in the United States in both categories (72% and 71%, respectively). The most common topic of study was cerebrovascular on both lists, followed by trauma on the most cited list vs. tumor on the most relevant list. The most relevant list also contained considerably more articles with a higher level of evidence such as systemic reviews/meta-analyses and prospective studies.

Conclusions

This study highlights the key contributing factors to the growth and flourishing of JNS. It also reveals several discrepancies between the most cited and most relevant articles, with the latter including more recently published articles, more studies addressing tumor, and more level I/1 (NHMRC/CEBM) evidence. Bibliometric analysis serves as a useful tool for clinicians and researchers to appraise published literature and understand the scientific foundation of modern neurosurgery.

## Introduction

The Journal of Neurosurgery (JNS), the official journal of the American Association of Neurological Surgeons (AANS) since 1944, has been dedicated to publishing state-of-the-art work relating to neurosurgery, which include but are not limited to clinical studies, laboratory investigations, technical notes, literature and systematic reviews, and expert opinions [1]. Numerous landmark articles that shape the scope and practice of neurosurgeons nowadays are published in JNS. Its leadership in the field of scientific publication has been acknowledged worldwide, currently as the top neurosurgical journal with an impact factor of 4.318 according to the latest Thomson Reuters Journal Citation Reports (JCR) [2].

With over 65 years of growth and maturation, JNS has witnessed the foundation and development of modern neurosurgery. Hence it is important not only to evaluate the academic significance of the articles published in JNS, but also seek their unifying elements. The number of citations of individual articles reflects its academic importance and impetus to encourage changes in practice or to lead future studies. Bibliometric analysis is therefore commonly applied to appraise the academic influence of journals and the articles therein [3-6]. Specifically, single journal analysis (of top-cited articles) serves as a useful tool to examine the objective performance and development of the journal [6-8] and thus is of significance to JNS as well. With the ranking of articles according to total citation counts, this would enable us to identify classic and foundational works and appraise their impact in their related fields.

However, an acknowledged limitation of using the total number of citations as a measure of impact is that older publications are favored over newer ones. Additionally, it is hard to determine the impact over time or the persisting significance of older publications by means of total citations. Previous analyses have overcome the bias of total citations by using an alternative measure of impact, i.e. the number of citations per year [3, 9]. This method may reveal the relevance of individual articles in modern practice by taking into account the effect of time. In light of this, we have performed a secondary citation analysis of articles published in JNS according to the number of citations per year.

The aim of this study was to determine and rank the 100 most cited (based on the total number of citations) and most relevant (based on the number of citations per year) articles originating in JNS respectively. Furthermore, the characteristics of the two rank lists were analyzed and compared regarding publication year, sources, (authorship, institution, country of origin) topic of article, type of article, and level of evidence.

## Materials and methods

Search strategy and data collection

The Web of Science database was searched on December 23, 2018 under “publication name” = Journal of Neurosurgery, with the inclusion of all article types and period 1944 to 2018. The results were ordered by “times cited” to yield a rank list of articles according to their numbers of total citations. The list of 100 most cited articles published in JNS was obtained with the ranking of total citations.

Since the database has not yet developed the function to sort results by “times cited per year”, we adopted the strategy of Bohl and Ponce’s study [3] by assuming that the top 100 articles ranked by number of citations per year would be captured within the list of the 1000 articles ranked by total number of citations. We then calculated the number of citations per year for each article using a similar method described previously [3]. Briefly, the number of months since publication was calculated from the publication month and year to the current month and year (December 2018). Articles without a month of publication were assumed to have been published in June. The number of years since publication was then calculated by dividing the months by 12. The number of citations per year was gained by dividing the total number of citations by the number of years since publication. The list of 100 most relevant articles published in JNS was thus generated by ranking with the number of citations per year.

The following information was further collected for each article from the rank lists: year of publication, sources (authorship, institution, country of origin) based on the corresponding author, of article (cerebrovascular, trauma, tumor, functional neurosurgery), type of article including clinical studies (retrospective, prospective, randomized controlled trials, case series, case reports), laboratory studies, reviews/meta-analysis, and guidelines/consensus statements. The level of evidence was determined according to the Australian National Health and Medical Research Council (NHMRC) and Oxford Centre for Evidence-based Medicine (CEBM) evidence hierarchy [10-11]. Articles of animal studies, laboratory studies, technical notes, and case reports were not included in such evidence hierarchy and thus were classified as level 0.

Statistical analysis

Comparisons between continuous data were done using ANOVA or Mann-Whitney U test according to the testing condition. Statistical significance was defined as p < 0.05. All of the tests were two-sided. Statistical analysis was performed using SPSS software (version 16.0, SPSS, Inc).

## Results

The 100 most cited articles published in JNS ranked by total number of citations are shown in Table 1. The median total number of citations was 505 (range 383-2200). The 100 most relevant articles published in JNS ranked by number of citations per year are shown in Table 2. The median number of citations per year was 21.88 (range 17.31-82.61). The most cited article was the Hunt-Hess grading scale for patients with subarachnoid hemorrhage (SAH) published in 1968 [12], while the most relevant article was the extent of resection and prognostic factors for survival in patients with glioblastoma multiforme (GBM) published by Lacroix et al. in 2001 [13]. 

**Table 1:** The 100 most cited articles from the Journal of Neurosurgery

Rank	Article	Total citations	Citations per year
1	Hunt WE, Hess RM: Surgical risk as related to time of intervention in the repair of intracranial aneurysms. J Neurosurg 28:14-20, 1968	2200	43.14
2	Aaslid R, Markwalder TM, Nornes H: Noninvasive transcranial Doppler ultrasound recording of flow velocity in basal cerebral arteries. J Neurosurg 57:769-774, 1982	2067	55.86
3	Lacroix M, Abi-Said D, Fourney DR, Gokaslan ZL, Shi W, DeMonte F, et al: A multivariate analysis of 416 patients with glioblastoma multiforme: prognosis, extent of resection, and survival. J Neurosurg 95:190-198, 2001	1487	82.61
4	Kassell NF, Torner JC, Haley EC, Jr., Jane JA, Adams HP, Kongable GL: The International Cooperative Study on the Timing of Aneurysm Surgery. Part 1: Overall management results. J Neurosurg 73:18-36, 1990	1391	47.97
5	Spetzler RF, Martin NA: A proposed grading system for arteriovenous malformations. J Neurosurg 65:476-483, 1986	1313	39.79
6	Walker MD, Alexander E, Jr., Hunt WE, MacCarty CS, Mahaley MS, Jr., Mealey J, Jr., et al: Evaluation of BCNU and/or radiotherapy in the treatment of anaplastic gliomas. A cooperative clinical trial. J Neurosurg 49:333-343, 1978	1276	31.12
7	Cloward RB: The anterior approach for removal of ruptured cervical disks. J Neurosurg 15:602-617, 1958	1136	18.62
8	Levy RM, Bredesen DE, Rosenblum ML: Neurological manifestations of the acquired immunodeficiency syndrome (AIDS): experience at UCSF and review of the literature. J Neurosurg 62:475-495, 1985	1084	31.88
9	Tator CH, Fehlings MG: Review of the secondary injury theory of acute spinal cord trauma with emphasis on vascular mechanisms. J Neurosurg 75:15-26, 1991	988	35.29
10	Ojemann G, Ojemann J, Lettich E, Berger M: Cortical language localization in left, dominant hemisphere. An electrical stimulation mapping investigation in 117 patients. J Neurosurg 71:316-326, 1989	973	32.43
11	Guglielmi G, Vinuela F, Dion J, Duckwiler G: Electrothrombosis of saccular aneurysms via endovascular approach. Part 2: Preliminary clinical experience. J Neurosurg 75:8-14, 1991	893	31.89
12	Marmarou A, Foda MA, van den Brink W, Campbell J, Kita H, Demetriadou K: A new model of diffuse brain injury in rats. Part I: Pathophysiology and biomechanics. J Neurosurg 80:291-300, 1994	883	35.32
13	Laitinen LV, Bergenheim AT, Hariz MI: Leksell's posteroventral pallidotomy in the treatment of Parkinson's disease. J Neurosurg 76:53-61, 1992	835	30.93
14	Jones TH, Morawetz RB, Crowell RM, Marcoux FW, FitzGibbon SJ, DeGirolami U, et al: Thresholds of focal cerebral ischemia in awake monkeys. J Neurosurg 54:773-782, 1981	832	21.89
15	Katayama Y, Becker DP, Tamura T, Hovda DA: Massive increases in extracellular potassium and the indiscriminate release of glutamate following concussive brain injury. J Neurosurg 73:889-900, 1990	785	27.07
16	Siesjo BK: Pathophysiology and treatment of focal cerebral ischemia. Part I: Pathophysiology. J Neurosurg 77:169-184, 1992	783	29
17	Dixon CE, Lyeth BG, Povlishock JT, Findling RL, Hamm RJ, Marmarou A, et al: A fluid percussion model of experimental brain injury in the rat. J Neurosurg 67:110-119, 1987	774	24.19
18	Benabid AL, Pollak P, Gao D, Hoffmann D, Limousin P, Gay E, et al: Chronic electrical stimulation of the ventralis intermedius nucleus of the thalamus as a treatment of movement disorders. J Neurosurg 84:203-214, 1996	759	33
19	Locksley HB: Natural history of subarachnoid hemorrhage, intracranial aneurysms and arteriovenous malformations. Based on 6368 cases in the cooperative study. J Neurosurg 25:219-239, 1966	752	14.19
20	Ondra SL, Troupp H, George ED, Schwab K: The natural history of symptomatic arteriovenous malformations of the brain: a 24-year follow-up assessment. J Neurosurg 73:387-391, 1990	719	24.79
21	Vinuela F, Duckwiler G, Mawad M: Guglielmi detachable coil embolization of acute intracranial aneurysm: perioperative anatomical and clinical outcome in 403 patients. J Neurosurg 86:475-482, 1997	717	32.59
22	Serbinenko FA: Balloon catheterization and occlusion of major cerebral vessels. J Neurosurg 41:125-145, 1974	707	15.71
23	Guglielmi G, Vinuela F, Sepetka I, Macellari V: Electrothrombosis of saccular aneurysms via endovascular approach. Part 1: Electrochemical basis, technique, and experimental results. J Neurosurg 75:1-7, 1991	706	25.21
24	Mirimanoff RO, Dosoretz DE, Linggood RM, Ojemann RG, Martuza RL: Meningioma: analysis of recurrence and progression following neurosurgical resection. J Neurosurg 62:18-24, 1985	690	20.29
25	Kassell NF, Torner JC, Jane JA, Haley EC, Jr., Adams HP: The International Cooperative Study on the Timing of Aneurysm Surgery. Part 2: Surgical results. J Neurosurg 73:37-47, 1990	686	23.66
26	Report of World Federation of Neurological Surgeons Committee on a Universal Subarachnoid Hemorrhage Grading Scale. J Neurosurg 68:985-986, 1988	676	21.81
27	Muizelaar JP, Marmarou A, Ward JD, Kontos HA, Choi SC, Becker DP, et al: Adverse effects of prolonged hyperventilation in patients with severe head injury: a randomized clinical trial. J Neurosurg 75:731-739, 1991	668	23.86
28	Becker DP, Miller JD, Ward JD, Greenberg RP, Young HF, Sakalas R: The outcome from severe head injury with early diagnosis and intensive management. J Neurosurg 47:491-502, 1977	660	15.71
29	Robinson JR, Awad IA, Little JR: Natural history of the cavernous angioma. J Neurosurg 75:709-714, 1991	643	22.96
30	Barrow DL, Spector RH, Braun IF, Landman JA, Tindall SC, Tindall GT: Classification and treatment of spontaneous carotid-cavernous sinus fistulas. J Neurosurg 62:248-256, 1985	636	18.71
31	Siesjo BK: Pathophysiology and treatment of focal cerebral ischemia. Part II: Mechanisms of damage and treatment. J Neurosurg 77:337-354, 1992	635	23.52
32	Rosner MJ, Rosner SD, Johnson AH: Cerebral perfusion pressure: management protocol and clinical results. J Neurosurg 83:949-962, 1995	627	26.13
33	Rivlin AS, Tator CH: Objective clinical assessment of motor function after experimental spinal cord injury in the rat. J Neurosurg 47:577-581, 1977	626	14.9
34	Levin HS, Mattis S, Ruff RM, Eisenberg HM, Marshall LF, Tabaddor K, et al: Neurobehavioral outcome following minor head injury: a three-center study. J Neurosurg 66:234-243, 1987	616	19.25
35	Obrist WD, Langfitt TW, Jaggi JL, Cruz J, Gennarelli TA: Cerebral blood flow and metabolism in comatose patients with acute head injury. Relationship to intracranial hypertension. J Neurosurg 61:241-253, 1984	606	17.31
36	Yasargil MG, Curcic M, Kis M, Siegenthaler G, Teddy PJ, Roth P: Total removal of craniopharyngiomas. Approaches and long-term results in 144 patients. J Neurosurg 73:3-11, 1990	588	20.28
37	Hochberg FH, Miller DC: Primary central nervous system lymphoma. J Neurosurg 68:835-853, 1988	582	18.77
38	Murayama Y, Nien YL, Duckwiler G, Gobin YP, Jahan R, Frazee J, et al: Guglielmi detachable coil embolization of cerebral aneurysms: 11 years' experience. J Neurosurg 98:959-966, 2003	568	35.5
39	Kelly PJ, Daumas-Duport C, Kispert DB, Kall BA, Scheithauer BW, Illig JJ: Imaging-based stereotaxic serial biopsies in untreated intracranial glial neoplasms. J Neurosurg 66:865-874, 1987	565	17.66
40	Jannetta PJ: Arterial compression of the trigeminal nerve at the pons in patients with trigeminal neuralgia. J Neurosurg 26:Suppl:159-162, 1967	563	10.83
41	Borden JA, Wu JK, Shucart WA: A proposed classification for spinal and cranial dural arteriovenous fistulous malformations and implications for treatment. J Neurosurg 82:166-179, 1995	552	23
42	Miller JD, Becker DP, Ward JD, Sullivan HG, Adams WE, Rosner MJ: Significance of intracranial hypertension in severe head injury. J Neurosurg 47:503-516, 1977	550	13.1
43	Perret G, Nishioka H: Report on the cooperative study of intracranial aneurysms and subarachnoid hemorrhage. Section VI. Arteriovenous malformations. An analysis of 545 cases of cranio-cerebral arteriovenous malformations and fistulae reported to the cooperative study. J Neurosurg 25:467-490, 1966	538	10.15
44	Jennings MT, Gelman R, Hochberg F: Intracranial germ-cell tumors: natural history and pathogenesis. J Neurosurg 63:155-167, 1985	531	15.62
45	Graf CJ, Perret GE, Torner JC: Bleeding from cerebral arteriovenous malformations as part of their natural history. J Neurosurg 58:331-337, 1983	530	14.72
46	Stummer W, Novotny A, Stepp H, Goetz C, Bise K, Reulen HJ: Fluorescence-guided resection of glioblastoma multiforme by using 5-aminolevulinic acid-induced porphyrins: a prospective study in 52 consecutive patients. J Neurosurg 93:1003-1013, 2000	527	27.74
47	Oldfield EH, Muraszko K, Shawker TH, Patronas NJ: Pathophysiology of syringomyelia associated with Chiari I malformation of the cerebellar tonsils. Implications for diagnosis and treatment. J Neurosurg 80:3-15, 1994	525	21
48	Lunsford LD, Kondziolka D, Flickinger JC, Bissonette DJ, Jungreis CA, Maitz AH, et al: Stereotactic radiosurgery for arteriovenous malformations of the brain. J Neurosurg 75:512-524, 1991	525	18.75
49	Zabramski JM, Wascher TM, Spetzler RF, Johnson B, Golfinos J, Drayer BP, et al: The natural history of familial cavernous malformations: results of an ongoing study. J Neurosurg 80:422-432, 1994	518	20.72
50	Sanai N, Polley M-Y, McDermott MW, Parsa AT, Berger MS: An extent of resection threshold for newly diagnosed glioblastomas. J Neurosurg 115:3-8, 2011	507	63.38
51	Bouma GJ, Muizelaar JP, Choi SC, Newlon PG, Young HF: Cerebral circulation and metabolism after severe traumatic brain injury: the elusive role of ischemia. J Neurosurg 75:685-693, 1991	504	18
52	Awad IA, Little JR, Akarawi WP, Ahl J: Intracranial dural arteriovenous malformations: factors predisposing to an aggressive neurological course. J Neurosurg 72:839-850, 1990	504	17.38
53	Backlund EO, Granberg PO, Hamberger B, Knutsson E, Martensson A, Sedvall G, et al: Transplantation of adrenal medullary tissue to striatum in parkinsonism. First clinical trials. J Neurosurg 62:169-173, 1985	503	14.79
54	Rorke LB, Packer RJ, Biegel JA: Central nervous system atypical teratoid/rhabdoid tumors of infancy and childhood: definition of an entity. J Neurosurg 85:56-65, 1996	501	21.78
55	Bracken MB, Shepard MJ, Collins WF, Jr., Holford TR, Baskin DS, Eisenberg HM, et al: Methylprednisolone or naloxone treatment after acute spinal cord injury: 1-year follow-up data. Results of the second National Acute Spinal Cord Injury Study. J Neurosurg 76:23-31, 1992	497	18.41
56	Jho HD, Carrau RL: Endoscopic endonasal transsphenoidal surgery: experience with 50 patients. J Neurosurg 87:44-51, 1997	481	21.86
57	Aaslid R, Huber P, Nornes H: Evaluation of cerebrovascular spasm with transcranial Doppler ultrasound. J Neurosurg 60:37-41, 1984	480	13.71
58	Woolsey CN, Erickson TC, Gilson WE: Localization in somatic sensory and motor areas of human cerebral cortex as determined by direct recording of evoked potentials and electrical stimulation. J Neurosurg 51:476-506, 1979	478	11.95
59	Guglielmi G, Vinuela F, Duckwiler G, Dion J, Lylyk P, Berenstein A, et al: Endovascular treatment of posterior circulation aneurysms by electrothrombosis using electrically detachable coils. J Neurosurg 77:515-524, 1992	470	17.41
60	Brown RD, Jr., Wiebers DO, Forbes G, O'Fallon WM, Piepgras DG, Marsh WR, et al: The natural history of unruptured intracranial arteriovenous malformations. J Neurosurg 68:352-357, 1988	469	15.13
61	Paddick I: A simple scoring ratio to index the conformity of radiosurgical treatment plans. Technical note. J Neurosurg 93 Suppl 3:219-222, 2000	467	24.58
62	Fahlbusch R, Honegger J, Paulus W, Huk W, Buchfelder M: Surgical treatment of craniopharyngiomas: experience with 168 patients. J Neurosurg 90:237-250, 1999	464	23.2
63	Duhaime AC, Gennarelli TA, Thibault LE, Bruce DA, Margulies SS, Wiser R: The shaken baby syndrome. A clinical, pathological, and biomechanical study. J Neurosurg 66:409-415, 1987	462	14.44
64	McCormick WF: The pathology of vascular ("arteriovenous") malformations. J Neurosurg 24:807-816, 1966	458	8.64
65	Matsutani M, Sano K, Takakura K, Fujimaki T, Nakamura O, Funata N, et al: Primary intracranial germ cell tumors: a clinical analysis of 153 histologically verified cases. J Neurosurg 86:446-455, 1997	457	20.77
66	Miller JD, Butterworth JF, Gudeman SK, Faulkner JE, Choi SC, Selhorst JB, et al: Further experience in the management of severe head injury. J Neurosurg 54:289-299, 1981	457	12.03
67	Fourney DR, Schomer DF, Nader R, Chlan-Fourney J, Suki D, Ahrar K, et al: Percutaneous vertebroplasty and kyphoplasty for painful vertebral body fractures in cancer patients. J Neurosurg 98:21-30, 2003	456	28.5
68	Jannetta PJ, Abbasy M, Maroon JC, Ramos FM, Albin MS: Etiology and definitive microsurgical treatment of hemifacial spasm. Operative techniques and results in 47 patients. J Neurosurg 47:321-328, 1977	454	10.81
69	Weir B, Grace M, Hansen J, Rothberg C: Time course of vasospasm in man. J Neurosurg 48:173-178, 1978	446	10.88
70	Steiner L, Lindquist C, Adler JR, Torner JC, Alves W, Steiner M: Clinical outcome of radiosurgery for cerebral arteriovenous malformations. J Neurosurg 77:1-8, 1992	441	16.33
71	Marmarou A, Shulman K, LaMorgese J: Compartmental analysis of compliance and outflow resistance of the cerebrospinal fluid system. J Neurosurg 43:523-534, 1975	438	9.95
72	Goldsmith BJ, Wara WM, Wilson CB, Larson DA: Postoperative irradiation for subtotally resected meningiomas. A retrospective analysis of 140 patients treated from 1967 to 1990. J Neurosurg 80:195-201, 1994	435	17.4
73	Lasjaunias P, Chiu M, ter Brugge K, Tolia A, Hurth M, Bernstein M: Neurological manifestations of intracranial dural arteriovenous malformations. J Neurosurg 64:724-730, 1986	435	13.18
74	McCormick PC, Torres R, Post KD, Stein BM: Intramedullary ependymoma of the spinal cord. J Neurosurg 72:523-532, 1990	434	14.97
75	Juvela S, Porras M, Poussa K: Natural history of unruptured intracranial aneurysms: probability of and risk factors for aneurysm rupture (Reprinted from J Neurosurg, vol 93, pg 379-387, 2000). J Neurosurg 108:1052-1060, 2008	432	39.27
76	McLaughlin MR, Jannetta PJ, Clyde BL, Subach BR, Comey CH, Resnick DK: Microvascular decompression of cranial nerves: lessons learned after 4400 operations. J Neurosurg 90:1-8, 1999	430	21.5
77	Kondziolka D, Lunsford LD, Kestle JR: The natural history of cerebral cavernous malformations. J Neurosurg 83:820-824, 1995	427	17.79
78	Del Curling O, Jr., Kelly DL, Jr., Elster AD, Craven TE: An analysis of the natural history of cavernous angiomas. J Neurosurg 75:702-708, 1991	427	15.25
79	Laws ER, Parney IF, Huang W, Anderson F, Morris AM, Asher A, et al: Survival following surgery and prognostic factors for recently diagnosed malignant glioma: data from the Glioma Outcomes Project. J Neurosurg 99:467-473, 2003	426	26.63
80	Roberts DW, Strohbehn JW, Hatch JF, Murray W, Kettenberger H: A frameless stereotaxic integration of computerized tomographic imaging and the operating microscope. J Neurosurg 65:545-549, 1986	426	12.91
81	Evans AE, Jenkin RD, Sposto R, Ortega JA, Wilson CB, Wara W, et al: The treatment of medulloblastoma. Results of a prospective randomized trial of radiation therapy with and without CCNU, vincristine, and prednisone. J Neurosurg 72:572-582, 1990	423	14.59
82	Simpkins JW, Rajakumar G, Zhang YQ, Simpkins CE, Greenwald D, Yu CJ, et al: Estrogens may reduce mortality and ischemic damage caused by middle cerebral artery occlusion in the female rat. J Neurosurg 87:724-730, 1997	419	19.05
83	Rigamonti D, Drayer BP, Johnson PC, Hadley MN, Zabramski J, Spetzler RF: The MRI appearance of cavernous malformations (angiomas). J Neurosurg 67:518-524, 1987	419	13.09
84	Siesjo BK: Cerebral circulation and metabolism. J Neurosurg 60:883-908, 1984	418	11.94
85	Laws ER, Jr., Taylor WF, Clifton MB, Okazaki H: Neurosurgical management of low-grade astrocytoma of the cerebral hemispheres. J Neurosurg 61:665-673, 1984	417	11.91
86	Locksley HB: Natural history of subarachnoid hemorrhage, intracranial aneurysms and arteriovenous malformations. J Neurosurg 25:321-368, 1966	415	7.83
87	Nashold BS, Jr., Wilson WP, Slaughter DG: Sensations evoked by stimulation in the midbrain of man. J Neurosurg 30:14-24, 1969	413	8.26
88	Shiozaki T, Sugimoto H, Taneda M, Yoshida H, Iwai A, Yoshioka T, et al: Effect of mild hypothermia on uncontrollable intracranial hypertension after severe head injury. J Neurosurg 79:363-368, 1993	408	15.69
89	Auer LM, Deinsberger W, Niederkorn K, Gell G, Kleinert R, Schneider G, et al: Endoscopic surgery versus medical treatment for spontaneous intracerebral hematoma: a randomized study. J Neurosurg 70:530-535, 1989	407	13.57
90	Narayan RK, Greenberg RP, Miller JD, Enas GG, Choi SC, Kishore PR, et al: Improved confidence of outcome prediction in severe head injury. A comparative analysis of the clinical examination, multimodality evoked potentials, CT scanning, and intracranial pressure. J Neurosurg 54:751-762, 1981	407	10.71
91	Eisenberg HM, Gary HE, Jr., Aldrich EF, Saydjari C, Turner B, Foulkes MA, et al: Initial CT findings in 753 patients with severe head injury. A report from the NIH Traumatic Coma Data Bank. J Neurosurg 73:688-698, 1990	405	13.97
92	Bouma GJ, Muizelaar JP, Stringer WA, Choi SC, Fatouros P, Young HF: Ultra-early evaluation of regional cerebral blood flow in severely head-injured patients using xenon-enhanced computerized tomography. J Neurosurg 77:360-368, 1992	402	14.89
93	Luerssen TG, Klauber MR, Marshall LF: Outcome from head injury related to patient's age. A longitudinal prospective study of adult and pediatric head injury. J Neurosurg 68:409-416, 1988	396	12.77
94	Gennarelli TA, Spielman GM, Langfitt TW, Gildenberg PL, Harrington T, Jane JA, et al: Influence of the type of intracranial lesion on outcome from severe head injury. J Neurosurg 56:26-32, 1982	395	10.68
95	Byrne JV, Sohn MJ, Molyneux AJ, Chir B: Five-year experience in using coil embolization for ruptured intracranial aneurysms: outcomes and incidence of late rebleeding. J Neurosurg 90:656-663, 1999	390	19.5
96	Marshall LF, Smith RW, Shapiro HM: The outcome with aggressive treatment in severe head injuries. Part I: the significance of intracranial pressure monitoring. J Neurosurg 50:20-25, 1979	390	9.75
97	Madawi AA, Casey AT, Solanki GA, Tuite G, Veres R, Crockard HA: Radiological and anatomical evaluation of the atlantoaxial transarticular screw fixation technique. J Neurosurg 86:961-968, 1997	388	17.64
98	Marmarou A, Shulman K, Rosende RM: A nonlinear analysis of the cerebrospinal fluid system and intracranial pressure dynamics. J Neurosurg 48:332-344, 1978	386	9.41
99	Marion DW, Obrist WD, Carlier PM, Penrod LE, Darby JM: The use of moderate therapeutic hypothermia for patients with severe head injuries: a preliminary report. J Neurosurg 79:354-362, 1993	385	14.81
100	Foda MA, Marmarou A: A new model of diffuse brain injury in rats. Part II: Morphological characterization. J Neurosurg 80:301-313, 1994	383	15.32

**Table 2:** The 100 most relevant articles from the Journal of Neurosurgery

Rank	Article	Total citations	Citations per year
1	Lacroix M, Abi-Said D, Fourney DR, Gokaslan ZL, Shi W, DeMonte F, et al: A multivariate analysis of 416 patients with glioblastoma multiforme: prognosis, extent of resection, and survival. J Neurosurg 95:190-198, 2001	1487	82.61
2	Sanai N, Polley M-Y, McDermott MW, Parsa AT, Berger MS: An extent of resection threshold for newly diagnosed glioblastomas. J Neurosurg 115:3-8, 2011	507	63.38
3	Aaslid R, Markwalder TM, Nornes H: Noninvasive transcranial Doppler ultrasound recording of flow velocity in basal cerebral arteries. J Neurosurg 57:769-774, 1982	2067	55.86
4	Kassell NF, Torner JC, Haley EC, Jr., Jane JA, Adams HP, Kongable GL: The International Cooperative Study on the Timing of Aneurysm Surgery. Part 1: Overall management results. J Neurosurg 73:18-36, 1990	1391	47.97
5	Hunt WE, Hess RM: Surgical risk as related to time of intervention in the repair of intracranial aneurysms. J Neurosurg 28:14-20, 1968	2200	43.14
6	Spetzler RF, Martin NA: A proposed grading system for arteriovenous malformations. J Neurosurg 65:476-483, 1986	1313	39.79
7	Juvela S, Porras M, Poussa K: Natural history of unruptured intracranial aneurysms: probability of and risk factors for aneurysm rupture (Reprinted from J Neurosurg, vol 93, pg 379-387, 2000). J Neurosurg 108:1052-1060, 2008	432	39.27
8	Murayama Y, Nien YL, Duckwiler G, Gobin YP, Jahan R, Frazee J, et al: Guglielmi detachable coil embolization of cerebral aneurysms: 11 years' experience. J Neurosurg 98:959-966, 2003	568	35.5
9	Marmarou A, Foda MA, van den Brink W, Campbell J, Kita H, Demetriadou K: A new model of diffuse brain injury in rats. Part I: Pathophysiology and biomechanics. J Neurosurg 80:291-300, 1994	883	35.32
10	Tator CH, Fehlings MG: Review of the secondary injury theory of acute spinal cord trauma with emphasis on vascular mechanisms. J Neurosurg 75:15-26, 1991	988	35.29
11	McGirt MJ, Chaichana KL, Gathinji M, Attenello FJ, Than K, Olivi A, et al: Independent association of extent of resection with survival in patients with malignant brain astrocytoma. Journal of Neurosurgery 110:156-162, 2009	352	35.2
12	Kassam AB, Preveoello DM, Carrau RL, Snyderman CH, Thomas A, Gardner P, et al: Endoscopic endonasal skull base surgery: analysis of complications in the authors' initial 800 patients. Journal of Neurosurgery 114:1544-1568, 2011	280	35
13	McDougall CG, Spetzler RF, Zabramski JM, Partovi S, Hills NK, Nakaji P, et al: The Barrow Ruptured Aneurysm Trial. Journal of Neurosurgery 116:135-144, 2012	243	34.71
14	Zhang Y, Chopp M, Meng Y, Katakowski M, Xin H, Mahmood A, et al: Effect of exosomes derived from multipluripotent mesenchyrnal stromal cells on functional recovery and neurovascular plasticity in rats after traumatic brain injury. Journal of Neurosurgery 122:856-867, 2015	137	34.25
15	Benabid AL, Pollak P, Gao D, Hoffmann D, Limousin P, Gay E, et al: Chronic electrical stimulation of the ventralis intermedius nucleus of the thalamus as a treatment of movement disorders. J Neurosurg 84:203-214, 1996	759	33
16	Vinuela F, Duckwiler G, Mawad M: Guglielmi detachable coil embolization of acute intracranial aneurysm: perioperative anatomical and clinical outcome in 403 patients. J Neurosurg 86:475-482, 1997	717	32.59
17	Ojemann G, Ojemann J, Lettich E, Berger M: Cortical language localization in left, dominant hemisphere. An electrical stimulation mapping investigation in 117 patients. J Neurosurg 71:316-326, 1989	973	32.43
18	Guglielmi G, Vinuela F, Dion J, Duckwiler G: Electrothrombosis of saccular aneurysms via endovascular approach. Part 2: Preliminary clinical experience. J Neurosurg 75:8-14, 1991	893	31.89
19	Levy RM, Bredesen DE, Rosenblum ML: Neurological manifestations of the acquired immunodeficiency syndrome (AIDS): experience at UCSF and review of the literature. J Neurosurg 62:475-495, 1985	1084	31.88
20	Walker MD, Alexander E, Jr., Hunt WE, MacCarty CS, Mahaley MS, Jr., Mealey J, Jr., et al: Evaluation of BCNU and/or radiotherapy in the treatment of anaplastic gliomas. A cooperative clinical trial. J Neurosurg 49:333-343, 1978	1276	31.12
21	Laitinen LV, Bergenheim AT, Hariz MI: Leksell's posteroventral pallidotomy in the treatment of Parkinson's disease. J Neurosurg 76:53-61, 1992	835	30.93
22	Gross BA, Du R: Natural history of cerebral arteriovenous malformations: a meta-analysis. Journal of Neurosurgery 118:437-443, 2013	183	30.5
23	Siesjo BK: Pathophysiology and treatment of focal cerebral ischemia. Part I: Pathophysiology. J Neurosurg 77:169-184, 1992	783	29
24	Fourney DR, Schomer DF, Nader R, Chlan-Fourney J, Suki D, Ahrar K, et al: Percutaneous vertebroplasty and kyphoplasty for painful vertebral body fractures in cancer patients. J Neurosurg 98:21-30, 2003	456	28.5
25	Aarabi B, Hesdorffer DC, Ahn ES, Aresco C, Scalea TM, Eisenberg HM: Outcome following decompressive craniectomy for malignant swelling due to severe head injury. Journal of neurosurgery 104:469-479, 2006	365	28.08
26	Stummer W, Novotny A, Stepp H, Goetz C, Bise K, Reulen HJ: Fluorescence-guided resection of glioblastoma multiforme by using 5-aminolevulinic acid-induced porphyrins: a prospective study in 52 consecutive patients. J Neurosurg 93:1003-1013, 2000	527	27.74
27	Katayama Y, Becker DP, Tamura T, Hovda DA: Massive increases in extracellular potassium and the indiscriminate release of glutamate following concussive brain injury. J Neurosurg 73:889-900, 1990	785	27.07
28	Laws ER, Parney IF, Huang W, Anderson F, Morris AM, Asher A, et al: Survival following surgery and prognostic factors for recently diagnosed malignant glioma: data from the Glioma Outcomes Project. J Neurosurg 99:467-473, 2003	426	26.63
29	Rosner MJ, Rosner SD, Johnson AH: Cerebral perfusion pressure: management protocol and clinical results. J Neurosurg 83:949-962, 1995	627	26.13
30	Farquharson S, Tournier JD, Calamante F, Fabinyi G, Schneider-Kolsky M, Jackson GD, et al: White matter fiber tractography: why we need to move beyond DTI. Journal of Neurosurgery 118:1367-1377, 2013	152	25.33
31	Guglielmi G, Vinuela F, Sepetka I, Macellari V: Electrothrombosis of saccular aneurysms via endovascular approach. Part 1: Electrochemical basis, technique, and experimental results. J Neurosurg 75:1-7, 1991	706	25.21
32	Ondra SL, Troupp H, George ED, Schwab K: The natural history of symptomatic arteriovenous malformations of the brain: a 24-year follow-up assessment. J Neurosurg 73:387-391, 1990	719	24.79
33	Inglese M, Makani S, Johnson G, Cohen BA, Silver JA, Gonen O, et al: Diffuse axonal injury in mild traumatic brain injury: a diffusion tensor imaging study. Journal of neurosurgery 103:298-303, 2005	345	24.64
34	Duffau H, Gatignol P, Mandonnet E, Capelle L, Taillandier L: Intraoperative subcortical stimulation mapping of language pathways in a consecutive series of 115 patients with Grade II glioma in the left dominant hemisphere. Journal of Neurosurgery 109:461-471, 2008	271	24.64
35	Paddick I: A simple scoring ratio to index the conformity of radiosurgical treatment plans. Technical note. J Neurosurg 93 Suppl 3:219-222, 2000	467	24.58
36	Dixon CE, Lyeth BG, Povlishock JT, Findling RL, Hamm RJ, Marmarou A, et al: A fluid percussion model of experimental brain injury in the rat. J Neurosurg 67:110-119, 1987	774	24.19
37	Lozano AM, Giacobbe P, Hamani C, Rizvi SJ, Kennedy SH, Kolivakis TT, et al: A multicenter pilot study of subcallosal cingulate area deep brain stimulation for treatment-resistant depression. Journal of Neurosurgery 116:315-322, 2012	168	24
38	Muizelaar JP, Marmarou A, Ward JD, Kontos HA, Choi SC, Becker DP, et al: Adverse effects of prolonged hyperventilation in patients with severe head injury: a randomized clinical trial. J Neurosurg 75:731-739, 1991	668	23.86
39	Kassell NF, Torner JC, Jane JA, Haley EC, Jr., Adams HP: The International Cooperative Study on the Timing of Aneurysm Surgery. Part 2: Surgical results. J Neurosurg 73:37-47, 1990	686	23.66
40	Siesjo BK: Pathophysiology and treatment of focal cerebral ischemia. Part II: Mechanisms of damage and treatment. J Neurosurg 77:337-354, 1992	635	23.52
41	Chang EF, Potts MB, Keles GE, Lamborn KR, Chang SM, Barbaro NM, et al: Seizure characteristics and control following resection in 332 patients with low-grade gliomas. Journal of Neurosurgery 108:227-235, 2008	258	23.45
42	Fahlbusch R, Honegger J, Paulus W, Huk W, Buchfelder M: Surgical treatment of craniopharyngiomas: experience with 168 patients. J Neurosurg 90:237-250, 1999	464	23.2
43	Borden JA, Wu JK, Shucart WA: A proposed classification for spinal and cranial dural arteriovenous fistulous malformations and implications for treatment. J Neurosurg 82:166-179, 1995	552	23
44	Raabe A, Nakaji P, Beck J, Kim LJ, Hsu FPK, Kamerman JD, et al: Prospective evaluation of surgical microscope-integrated intraoperative near-infrared indocyanine green videoangiography during aneurysm surgery. Journal of neurosurgery 103:982-989, 2005	322	23
45	Robinson JR, Awad IA, Little JR: Natural history of the cavernous angioma. J Neurosurg 75:709-714, 1991	643	22.96
46	Bailes JE, Petraglia AL, Omalu BI, Nauman E, Talavage T: Role of subconcussion in repetitive mild traumatic brain injury. Journal of Neurosurgery 119:1235-1245, 2013	136	22.67
47	Cameron T: Safety and efficacy of spinal cord stimulation for the treatment of chronic pain: a 20-year literature review. Journal of neurosurgery 100:254-267, 2004	339	22.6
48	Slevin JT, Gerhardt GA, Smith CD, Gash DM, Kryscio R, Young B: Improvement of bilateral motor functions in patients with Parkinson disease through the unilateral intraputaminal infusion of glial cell line-derived neurotrophic factor. Journal of neurosurgery 102:216-222, 2005	314	22.43
49	Knisely JPS, Yu JB, Flanigan J, Sznol M, Kluger HM, Chiang VLS: Radiosurgery for melanoma brain metastases in the ipilimumab era and the possibility of longer survival. Journal of Neurosurgery 117:227-233, 2012	154	22
50	Jones TH, Morawetz RB, Crowell RM, Marcoux FW, FitzGibbon SJ, DeGirolami U, et al: Thresholds of focal cerebral ischemia in awake monkeys. J Neurosurg 54:773-782, 1981	832	21.89
51	Jho HD, Carrau RL: Endoscopic endonasal transsphenoidal surgery: experience with 50 patients. J Neurosurg 87:44-51, 1997	481	21.86
52	Report of World Federation of Neurological Surgeons Committee on a Universal Subarachnoid Hemorrhage Grading Scale. J Neurosurg 68:985-986, 1988	676	21.81
53	Rorke LB, Packer RJ, Biegel JA: Central nervous system atypical teratoid/rhabdoid tumors of infancy and childhood: definition of an entity. J Neurosurg 85:56-65, 1996	501	21.78
54	McLaughlin MR, Jannetta PJ, Clyde BL, Subach BR, Comey CH, Resnick DK: Microvascular decompression of cranial nerves: lessons learned after 4400 operations. J Neurosurg 90:1-8, 1999	430	21.5
55	Zrinzo L, Foltynie T, Limousin P, Hariz MI: Reducing hemorrhagic complications in functional neurosurgery: a large case series and systematic literature review. Journal of Neurosurgery 116:84-94, 2012	150	21.43
56	Oldfield EH, Muraszko K, Shawker TH, Patronas NJ: Pathophysiology of syringomyelia associated with Chiari I malformation of the cerebellar tonsils. Implications for diagnosis and treatment. J Neurosurg 80:3-15, 1994	525	21
57	Bloch O, Han SJ, Cha S, Sun MZ, Aghi MK, McDermott MW, et al: Impact of extent of resection for recurrent glioblastoma on overall survival. Journal of Neurosurgery 117:1032-1038, 2012	147	21
58	Matsutani M, Sano K, Takakura K, Fujimaki T, Nakamura O, Funata N, et al: Primary intracranial germ cell tumors: a clinical analysis of 153 histologically verified cases. J Neurosurg 86:446-455, 1997	457	20.77
59	Zabramski JM, Wascher TM, Spetzler RF, Johnson B, Golfinos J, Drayer BP, et al: The natural history of familial cavernous malformations: results of an ongoing study. J Neurosurg 80:422-432, 1994	518	20.72
60	Mirimanoff RO, Dosoretz DE, Linggood RM, Ojemann RG, Martuza RL: Meningioma: analysis of recurrence and progression following neurosurgical resection. J Neurosurg 62:18-24, 1985	690	20.29
61	Yasargil MG, Curcic M, Kis M, Siegenthaler G, Teddy PJ, Roth P: Total removal of craniopharyngiomas. Approaches and long-term results in 144 patients. J Neurosurg 73:3-11, 1990	588	20.28
62	Lovell MR, Collins MW, Iverson GL, Field M, Maroon JC, Cantu R, et al: Recovery from mild concussion in high school athletes. Journal of neurosurgery 98:296-301, 2003	324	20.25
63	Tabaee A, Anand VK, Barron Y, Hiltzik DH, Brown SM, Kacker A, et al: Endoscopic pituitary surgery: a systematic review and meta-analysis. Journal of Neurosurgery 111:545-554, 2009	201	20.1
64	Laufer I, Anand VK, Schwartz TH: Endoscopic, endonasal extended transsphenoidal, transplanum transtuberculum approach for resection of suprasellar lesions. Journal of Neurosurgery 106:400-406, 2007	241	20.08
65	Siddiqui AH, Abla AA, Kan P, Dumont TM, Jahshan S, Britz GW, et al: Panacea or problem: flow diverters in the treatment of symptomatic large or giant fusiform vertebrobasilar aneurysms. Journal of Neurosurgery 116:1258-1266, 2012	139	19.86
66	Hukkelhoven CWPM, Steyerberg EW, Rampen AJJ, Farace E, Habbema JDF, Marshall LF, et al: Patient age and outcome following severe traumatic brain injury: an analysis of 5600 patients. Journal of neurosurgery 99:666-673, 2003	316	19.75
67	Kondziolka D, Steinberg GK, Wechsler L, Meltzer CC, Elder E, Gebel J, et al: Neurotransplantation for patients with subcortical motor stroke: a phase 2 randomized trial. Journal of neurosurgery 103:38-45, 2005	275	19.64
68	Yordanova YN, Moritz-Gasser S, Duffau H: Awake surgery for WHO Grade II gliomas within "noneloquent" areas in the left dominant hemisphere: toward a "supratotal" resection. Journal of Neurosurgery 115:232-239, 2011	157	19.63
69	Huang F-P, Xi G, Keep RF, Hua Y, Nemoianu A, Hoff JT: Brain edema after experimental intracerebral hemorrhage: role of hemoglobin degradation products. Journal of neurosurgery 96:287-293, 2002	332	19.53
70	Byrne JV, Sohn MJ, Molyneux AJ, Chir B: Five-year experience in using coil embolization for ruptured intracranial aneurysms: outcomes and incidence of late rebleeding. J Neurosurg 90:656-663, 1999	390	19.5
71	Scott RM, Smith JL, Robertson RL, Madsen JR, Soriano SG, Rockoff MA: Long-term outcome in children with moyamoya syndrome after cranial revascularization by pial synangiosis. Journal of neurosurgery 100:142-149, 2004	292	19.47
72	Levin HS, Mattis S, Ruff RM, Eisenberg HM, Marshall LF, Tabaddor K, et al: Neurobehavioral outcome following minor head injury: a three-center study. J Neurosurg 66:234-243, 1987	616	19.25
73	Simpkins JW, Rajakumar G, Zhang YQ, Simpkins CE, Greenwald D, Yu CJ, et al: Estrogens may reduce mortality and ischemic damage caused by middle cerebral artery occlusion in the female rat. J Neurosurg 87:724-730, 1997	419	19.05
74	Duffau H, Capelle L, Denvil D, Sichez N, Gatignol P, Taillandier L, et al: Usefulness of intraoperative electrical subcortical mapping during surgery for low-grade gliomas located within eloquent brain regions: functional results in a consecutive series of 103 patients. Journal of neurosurgery 98:764-778, 2003	304	19
75	Claus EB, Bondy ML, Schildkraut JM, Wiemels JL, Wrensch M, Black PM: Epidemiology of intracranial meningioma. Neurosurgery 57:1088-1095; discussion 1088-1095, 2005	266	19
76	Higashida T, Kreipke CW, Rafols JA, Peng C, Schafer S, Schafer P, et al: The role of hypoxia-inducible factor-la, aquaporin-4, and matrix metalloproteinase-9 in blood-brain barrier disruption and brain edema after traumatic brain injury. Journal of Neurosurgery 114:92-101, 2011	152	19
77	Cappabianca P, Cavallo LM, Colao A, de Divitiis E: Surgical complications associated with the endoscopic endonasal transsphenoidal approach for pituitary adenomas. Journal of neurosurgery 97:293-298, 2002	321	18.88
78	Hochberg FH, Miller DC: Primary central nervous system lymphoma. J Neurosurg 68:835-853, 1988	582	18.77
79	Lunsford LD, Kondziolka D, Flickinger JC, Bissonette DJ, Jungreis CA, Maitz AH, et al: Stereotactic radiosurgery for arteriovenous malformations of the brain. J Neurosurg 75:512-524, 1991	525	18.75
80	Saatci I, Geyik S, Yavuz K, Cekirge HS: Endovascular treatment of brain arteriovenous malformations with prolonged intranidal Onyx injection technique: long-term results in 350 consecutive patients with completed endovascular treatment course. Journal of Neurosurgery 115:78-88, 2011	150	18.75
81	Barrow DL, Spector RH, Braun IF, Landman JA, Tindall SC, Tindall GT: Classification and treatment of spontaneous carotid-cavernous sinus fistulas. J Neurosurg 62:248-256, 1985	636	18.71
82	Cloward RB: The anterior approach for removal of ruptured cervical disks. J Neurosurg 15:602-617, 1958	1136	18.62
83	Bracken MB, Shepard MJ, Collins WF, Jr., Holford TR, Baskin DS, Eisenberg HM, et al: Methylprednisolone or naloxone treatment after acute spinal cord injury: 1-year follow-up data. Results of the second National Acute Spinal Cord Injury Study. J Neurosurg 76:23-31, 1992	497	18.41
84	Kassam AB, Gardner PA, Snyderman CH, Carrau RL, Mintz AH, Prevedello DM: Expanded endonasal approach, a fully endoscopic transnasal approach for the resection of midline suprasellar craniopharyngiomas: a new classification based on the infundibulum. Journal of Neurosurgery 108:715-728, 2008	201	18.27
85	Englot DJ, Chang EF, Auguste KI: Vagus nerve stimulation for epilepsy: a meta-analysis of efficacy and predictors of response. Journal of Neurosurgery 115:1248-1255, 2011	146	18.25
86	Starr PA, Christine CW, Theodosopoulos PV, Lindsey N, Byrd D, Mosley A, et al: Implantation of deep brain stimulators into the subthalamic nucleus: technical approach and magnetic resonance imaging-verified lead locations. Journal of neurosurgery 97:370-387, 2002	310	18.24
87	Bouma GJ, Muizelaar JP, Choi SC, Newlon PG, Young HF: Cerebral circulation and metabolism after severe traumatic brain injury: the elusive role of ischemia. J Neurosurg 75:685-693, 1991	504	18
88	Guzman R, Lee M, Achrol A, Bell-Stephens T, Kelly M, Do HM, et al: Clinical outcome after 450 revascularization procedures for moyamoya disease. Journal of Neurosurgery 111:927-935, 2009	180	18
89	Lunsford LD, Niranjan A, Flickinger JC, Maitz A, Kondziolka D: Radiosurgery of vestibular schwannomas: summary of experience in 829 cases. Journal of neurosurgery 102:195-199, 2005	251	17.93
90	Davis FG, Freels S, Grutsch J, Barlas S, Brem S: Survival rates in patients with primary malignant brain tumors stratified by patient age and tumor histological type: an analysis based on Surveillance, Epidemiology, and End Results (SEER) data, 1973-1991. Journal of neurosurgery 88:1-10, 1998	375	17.86
91	Kondziolka D, Lunsford LD, Kestle JR: The natural history of cerebral cavernous malformations. J Neurosurg 83:820-824, 1995	427	17.79
92	Kelly PJ, Daumas-Duport C, Kispert DB, Kall BA, Scheithauer BW, Illig JJ: Imaging-based stereotaxic serial biopsies in untreated intracranial glial neoplasms. J Neurosurg 66:865-874, 1987	565	17.66
93	Madawi AA, Casey AT, Solanki GA, Tuite G, Veres R, Crockard HA: Radiological and anatomical evaluation of the atlantoaxial transarticular screw fixation technique. J Neurosurg 86:961-968, 1997	388	17.64
94	Guglielmi G, Vinuela F, Duckwiler G, Dion J, Lylyk P, Berenstein A, et al: Endovascular treatment of posterior circulation aneurysms by electrothrombosis using electrically detachable coils. J Neurosurg 77:515-524, 1992	470	17.41
95	Goldsmith BJ, Wara WM, Wilson CB, Larson DA: Postoperative irradiation for subtotally resected meningiomas. A retrospective analysis of 140 patients treated from 1967 to 1990. J Neurosurg 80:195-201, 1994	435	17.4
96	Mocco J, Snyder KV, Albuquerque FC, Bendok BR, Boulos AS, Carpenter JS, et al: Treatment of intracranial aneurysms with the Enterprise stent: a multicenter registry. Journal of Neurosurgery 110:35-39, 2009	174	17.4
97	Keles GE, Lamborn KR, Berger MS: Low-grade hemispheric gliomas in adults: a critical review of extent of resection as a factor influencing outcome. Journal of neurosurgery 95:735-745, 2001	313	17.39
98	Awad IA, Little JR, Akarawi WP, Ahl J: Intracranial dural arteriovenous malformations: factors predisposing to an aggressive neurological course. J Neurosurg 72:839-850, 1990	504	17.38
99	Bergsneider M, Hovda DA, Shalmon E, Kelly DF, Vespa PM, Martin NA, et al: Cerebral hyperglycolysis following severe traumatic brain injury in humans: a positron emission tomography study. Journal of neurosurgery 86:241-251, 1997	382	17.36
100	Obrist WD, Langfitt TW, Jaggi JL, Cruz J, Gennarelli TA: Cerebral blood flow and metabolism in comatose patients with acute head injury. Relationship to intracranial hypertension. J Neurosurg 61:241-253, 1984	606	17.31

Characteristics of the most cited rank list are detailed as follows:

Temporal trends

**Figure 1:**
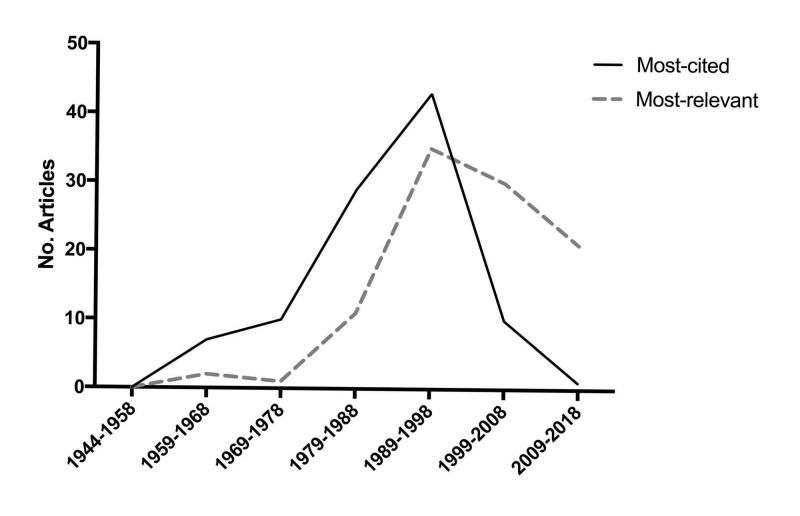
Trends in the 100 most cited vs. most relevant articles over time.

**Figure 2:**
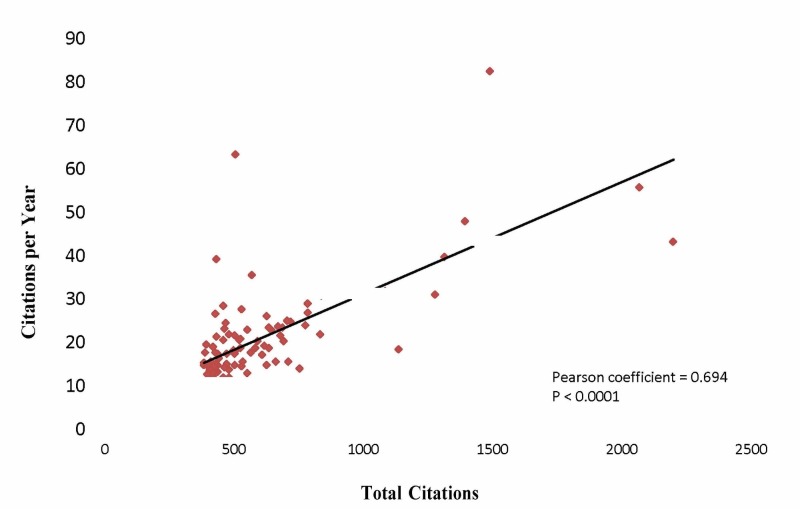
Positive correlation between the total number of citations and the number of citations per year.

**Figure 3:**
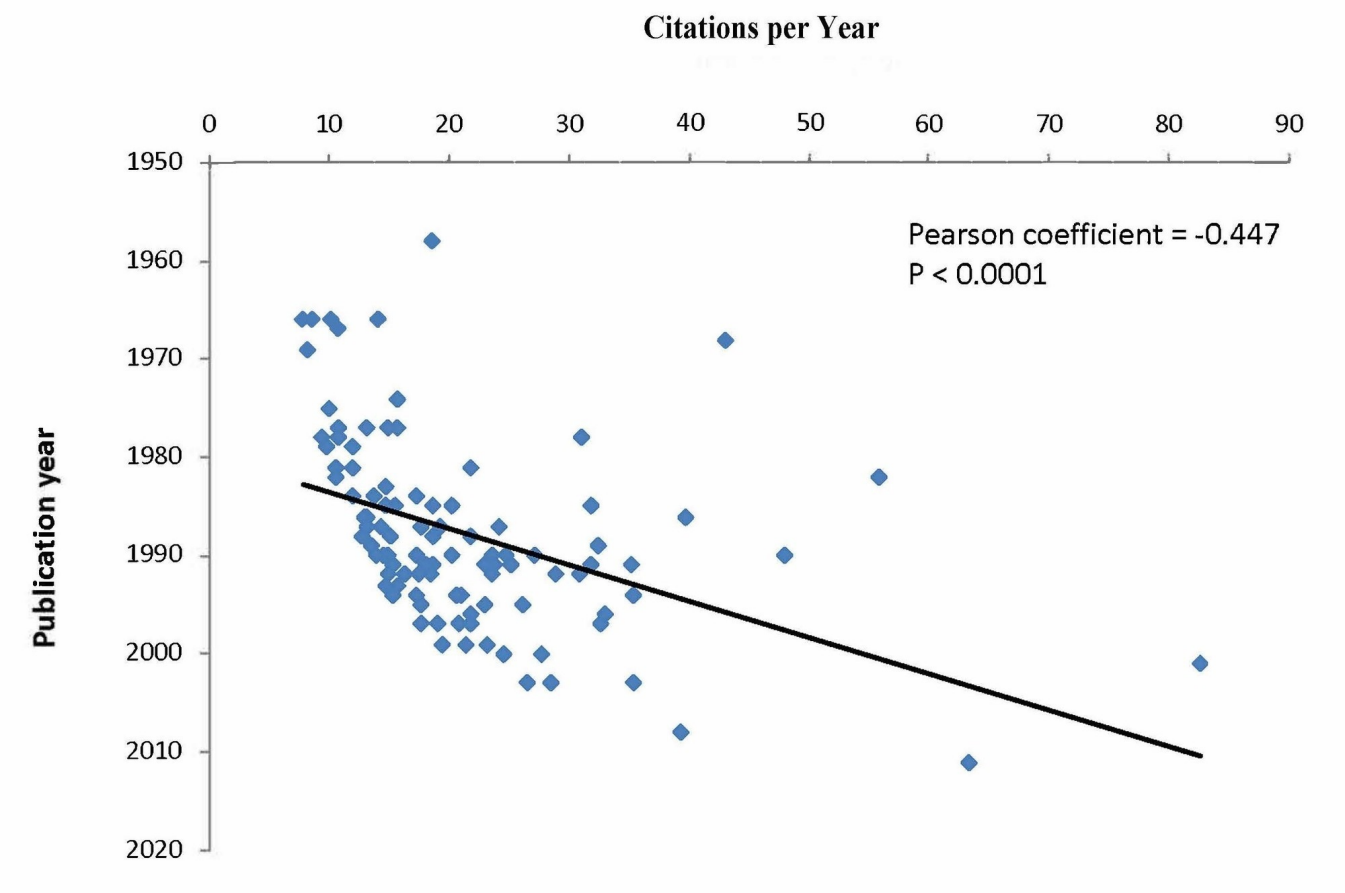
Negative correlation between the number of citations per year and the number of years since publication.

Figure 1 shows a sharp increase in the number of most cited articles in the late 1970s, with a decrease in the most recent decade. Seventy-five percent of all most cited articles were published between 1979 and 1999, with the 1990s as the apex (n=44). There was a positive correlation between the total number of citations and the number of citations per year (Pearson coefficient = 0.694, P < 0.0001) (Figure 2), whereas a negative correlation was found between the number of citations per year and the number of years since publication (Pearson coefficient = −0.447, P < 0.0001) (Figure 3).

Sources

North American institutions contributed to the majority of the top cited 100 articles (n=77), with Sweden following with five articles. The rest of the articles on the list originated from nine different countries including Switzerland, Italy, United Kingdom, Finland, Germany, Japan, France, Austria, and the former Soviet Union (Table 3). 

**Table 3:** Summary of the 100 most cited articles based on citation count vs. summary of the 100 most relevant articles

Variable	The 100 most cited articles								The 100 most relevant articles
	Citation count								
	Total	>2000	1001-2000	801-1000	601-800	501-600	401-500	301-400	Total
Total	100	2	6	6	21	19	38	8	100
Country of origin									
US	72	1	6	3	13	16	27	6	71
Canada	5	/	/	1	2	/	2	/	3
Sweden	5	/	/	1	2	1	1	/	3
Switzerland	3	1	/	/	/	1	1	/	2
Italy	3	/	/	1	1	/	1	/	4
UK	3	/	/	/	/	/	1	2	4
Finland	2	/	/	/	1	/	1	/	2
Germany	2	/	/	/	/	1	1	/	3
Japan	2	/	/	/	/	/	2	/	1
France	1	/	/	/	1	/	/	/	4
Others	2	/	/	/	1	/	1	/	3
Topic of article									
Cerebrovascular	41	2	2	2	12	7	15	1	36
Trauma	26	/	1	2	7	2	8	6	20
Tumor	18	/	2	/	1	7	8	/	30
Functional neurosurgery	11	/	/	2	1	2	6	/	12
Others	4	/	1	/	/	1	1	1	2
Type of article									
Clinical studies	81	2	5	3	15	18	32	6	76
Laboratory studies	12	/	/	2	4	/	4	2	10
Reviews/ meta-analysis	7	/	1	1	2	1	2	/	14
Guidelines/ consensus statements	/								/

**Figure 4:**
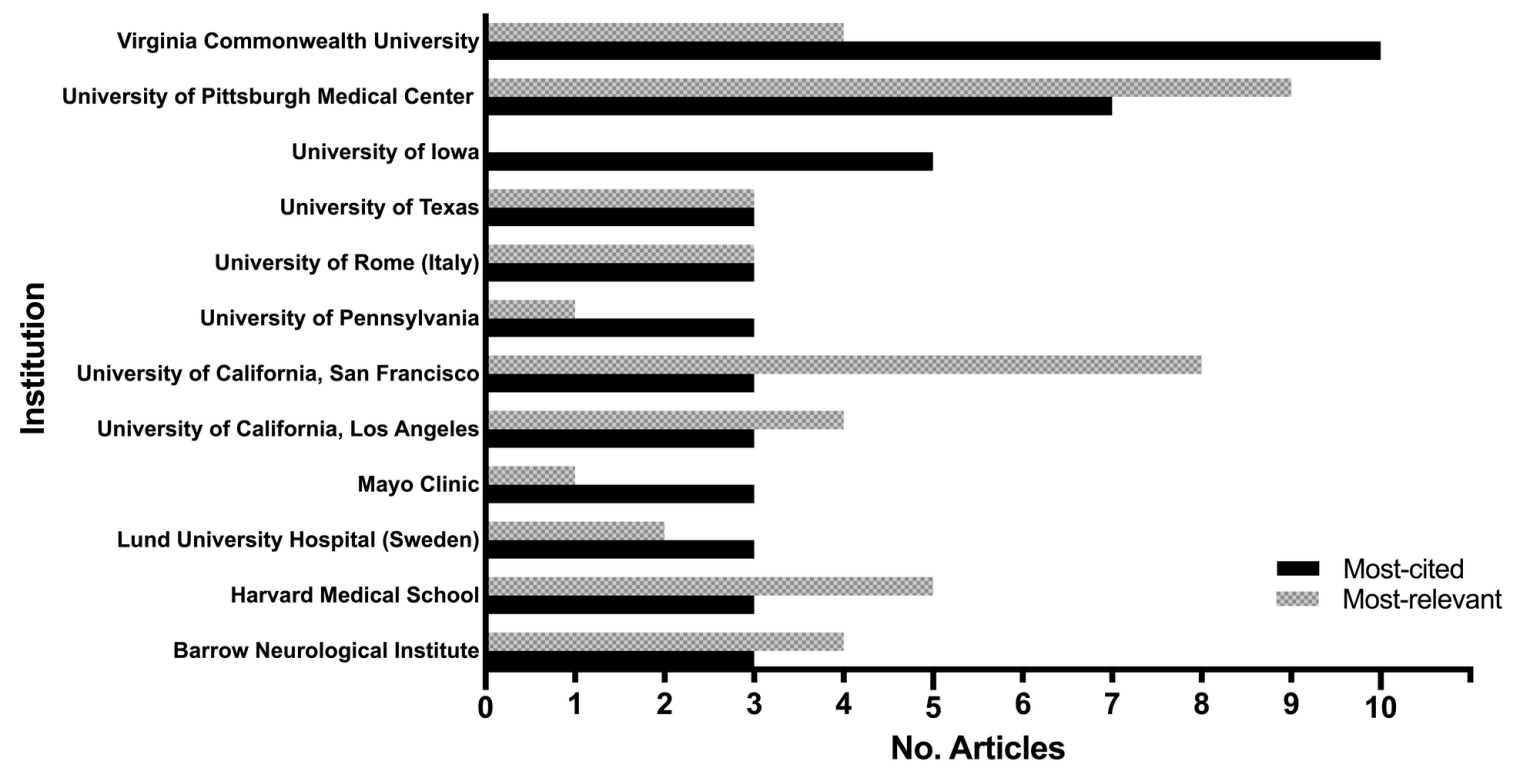
Institutions with corresponding author contributing three or more articles to the top 100 most cited and top 100 most relevant articles from the Journal of Neurosurgery.

The top institutions with corresponding author contributing three or more articles are shown in Figure 4. The Medical College of Virginia, of Virginia Commonwealth University, was the leading center on the top cited list, with 10 articles all addressing traumatic brain injury (TBI), followed by University of Pittsburgh Medical Center with seven articles. Lund University Hospital in Sweden and the University of Rome in Italy shared the most productive international institutions, with each contributing three articles.

Authors who contributed three or more articles to the top cited list are shown in Table 4 with their position on the author list. Anthony Marmarou and Harold F. Young shared the top of the most productive authors ranking contributing seven articles each. Anthony Marmarou and Guido Guglielmi contributed the highest number of research articles (n=3) as the first author, while Bo K. Siesjo contributed three review articles as first and sole author.

**Table 4:** Authors contributing three or more articles to the 100 most cited articles * Refers to the article number in Table 1 † Rank last in the author list as corresponding author

Name	No. of articles	Position on author list	Article Number*
Marmarou, Anthony	7	First (3), Second (1), Sixth (1), Eighth (1), Senior† (1)	12, 17, 27, 71, 91, 98, 100
Young, Harold F	7	Fifth (2), Sixth (1), Seventh (1), Eighth (1), Ninth (1), Tenth (1)	17, 27, 28, 51, 66, 91, 92
Becker, Donald P	6	First (1), Second (2), Sixth (1), Ninth (1), Tenth (1)	15, 27, 28, 42, 66, 90
Marshall, Lawrence F	6	First (1), Third (1), Fifth (1), Seventh (1), Ninth (1), Tenth (1)	34, 55, 91, 93, 94, 96
Miller, J Douglas	5	First (2), Second (1), Third (1), Eighth (1)	28, 42, 66, 90, 94
Vinuela, Fernando	5	First (1), Second (3), Eighth (1)	11, 21, 23, 38, 59
Choi, Sung C	5	Third (1), Fourth (1), Fifth (3)	27, 51, 66, 90, 92
Torner, James C	4	Second (2), Third (1), Fourth (1)	4, 25, 45, 70
Duckwiler, Gary	4	Second (1), Third (2), Fourth (1)	11, 21, 38, 59
Jane, John A	4	Third (1), Fourth (1), Sixth (1), Seventh (1)	4, 25, 91, 94
Guglielmi, Guido	3	First (3)	11, 23, 59
Siesjo, Bo K	3	First (3)	16, 31, 84
Jannetta, Peter J	3	First (2), Second (1)	40, 68, 76
Muizelaar, J Paul	3	First (1), Second (2)	27, 51, 92
Gennarelli, Thomas A	3	First (1), Second (1), Fifth (1)	35, 63, 94
Spetzler, Robert F	3	First (1), Third (1), Senior† (1)	5, 29, 83
Eisenberg, Howard M	3	First (1), Fourth (1), Sixth (1)	34, 55, 91
Ward, John D	3	Third (3)	27, 28, 42
Wilson, Charles B	3	Third (1), Fifth (1), Tenth (1)	6, 72, 81
Berger, Mitchel S	3	Fourth (1), Eleventh (1), Senior† (1)	10, 50, 79

Topic of article

Articles were classified into four categories including cerebrovascular, trauma, tumor, and functional neurosurgery. Ninety-six percent of articles fit into such classifications while four articles not classified by these categories were related to infection and cerebrospinal fluid system (numbers 8, 47, 71, and 98 in Table 1). A detailed breakdown of different study topics is shown in Table 5.

**Table 5:** The 100 most cited articles by topic AIDS = acquired immunodeficiency syndrome, AVM = arteriovenous malformation, CT= computed tomography, CCSF = carotid-cavernous sinus fistula, DBS = deep brain stimulation, IA = intracranial aneurysms, CSF = cerebrospinal fluid, ICP = intracranial pressure, ICU = intensive care unit, MRI = magnetic resonance imaging, MVD = Microvascular decompression, PCNSL = Primary central nervous system lymphoma, SAH = subarachnoid hemorrhage, SCI = spinal cord injury, SDAVF = spinal dural arteriovenous fistula, TBI = traumatic brain injury * Refers to the article number in Table 1

Topic of article	No. of articles	Article Number*
Cerebrovascular	41	
IA and/or SAH	16	
Endovascular treatment	7	11, 21, 22, 23, 38, 59, 95
Open surgery	4	1, 4, 25, 26
Natural history	4	19, 69, 75, 86
Endoscopic surgery for spontaneous SAH	1	89
Vascular malformations	17	
Intracranial AVM	9	5, 20, 45, 48, 52, 60, 64, 70, 73
Cavernous malformation	5	29, 49, 77, 78, 83
CCSF	1	30
SDAVF	1	41
Miscellaneous	1	43
Cerebral blood flow and ischemia	8	
Pathophysiology	4	14, 16, 31, 84
Treatment	2	32, 82
Transcranial Doppler study of blood flow	2	2, 57
Trauma	26	
Mild TBI: pathophysiology, outcome	2	15, 34
Severe TBI	13	
Monitoring of ICP and prognostic factors for outcome	6	42, 66, 90, 91, 94, 96
Cerebral circulation	3	35, 51, 92
ICU management and surgery	2	27, 28,
Therapeutic hypothermia	2	88, 99
SCI	6	
Pathophysiology	1	9
Anterior approach for ruptured cervical disks	1	7
Atlantoaxial transarticular screw fixation	1	97
Percutaneous vertebroplasty/kyphoplasty in cancer patients	1	67
Methylprednisolone/naloxone treatment for acute SCI	1	55
Motor function assessment in rat SCI model	1	33
Other	5	
Animal models	3	12, 17, 100
Prognostic factors for survival	1	93
Shaken baby syndrome	1	63
Tumor	18	
Glioma	7	
Extent of resection and prognostic factors for survival	5	3, 46, 50, 79, 85
Chemotherapy and/or radiation	1	6
Image-based stereotaxic biopsy	1	39
Sellar/parasellar tumors, transsphenoidal approaches	3	36, 56, 62
Meningioma: surgical outcome, postoperative irradiation	2	24, 72
Others	6	
Germ cell tumors	2	44, 65
PCNSL	1	37
Atypical teratoid/rhabdoid tumor	1	54
Ependymoma	1	74
Medulloblastoma	1	81
Functional neurosurgery	11	
Disorders		
Parkinson disease and other movement disorders	3	13, 18, 53
Trigeminal neuralgia, hemifacial spasm	3	40, 68, 76
Procedures		
DBS	1	18
Implantation of biological agents	1	53
Pallidotomy	1	13
MVD	3	40, 68, 76
Diagnostic studies	3	
Cortical language localization by electric stimulation mapping	1	10
Somatic sensory and motor localization by cortical evoked potentials	1	58
Sensory responses to electric stimulation of midbrain	1	87
Stereotaxy	2	
Frameless stereotaxy with integration of CT and microscope	1	80
Conformity index for radiosurgery	1	61
Other	4	
Neurologic manifestations of AIDS	1	8
Syringomyelia	1	47
Mathematical model of CSF system and ICP dynamics	2	71, 98

The most common topic of study was cerebrovascular (n=41). The best-represented subtopic was intracranial arteriovenous malformation (AVM)(n=9), followed by endovascular treatment for intracranial aneurysms (n=7).

Trauma studies represented the second most common topic (n=26), with the majority addressing traumatic brain injury (TBI), which included mild TBI (n=2), severe TBI (n=13), animal models (n=3), prognostic factors for survival (n=1), and shaken baby syndrome (n=1). The remaining six articles were related to spinal injury.

There were eighteen articles reporting tumor topics, of which seven addressed glioma. Of note, articles featuring the extent of surgical resection and other prognostic factors for survival represented the mainstay (n=5). Other tumor topics included studies on sellar/parasellar tumors, meningioma, and other less common tumors.

Articles on functional neurosurgery composed the least number of articles on the list of 100 most cited articles (n=11). Subtopics pertaining to functional neurosurgery included movement disorders (n=3), microvascular decompression (n=3), diagnostic studies with electrical stimulation (n=3), and stereotaxy (n=2).

Type of article and level of evidence

Articles were further classified according to the type of study into four categories which included clinical studies (retrospective, prospective, randomized controlled trials, case series, and case reports), laboratory studies, reviews/meta-analysis, and guidelines/consensus statements (Table 3). None of the articles were guidelines/consensus statements. There were 81 clinical studies, among which more than half were retrospective studies and case series (n=46) corresponding to level IV/4 (NHMRC/CEBM) evidence. There were twenty-six prospective studies and seven randomized controlled trials corresponding to level III/2 and II/1b (NHMRC/ CEBM) evidence, respectively. None of the articles were systematic review/meta-analysis, and thus there was no level I/1 (NHMRC/CEBM) evidence. Six literature review articles were level V/5 (NHMRC/CEBM) evidence. A detailed breakdown of different study types along with their level of evidence is shown in Table 6.

**Table 6:** The 100 most cited articles by type and level of evidence CEBM = Centre for Evidence-based Medicine, NHMRC = National Health and Medical Research Council * Refers to the article number in Table 1

Level of evidence		Type of article	No. of articles	Article Number*
NHMRC	CEBM			
I	1	Systematic review/Meta-analysis	0	
II	1b	Randomized controlled trial	7	6, 27, 55, 81, 88, 89, 99
III	2	Original prospective		
		Clinical	22	4, 18, 20, 21, 25, 26, 32, 34, 46, 49, 51, 53, 59, 66, 77, 79, 90, 91, 92, 93, 94, 95
		Experimental	4	2, 39, 47, 57
	3	Case-control study	2	24, 75
IV	4	Original retrospective study	21	1, 3, 5, 19, 28, 29, 38, 42, 43, 44, 45, 48, 50, 60, 65, 72, 78, 85, 86, 96, 97
		Case series	25	7, 10, 11, 13, 22, 30, 35, 36, 40, 41, 52, 54, 56, 58, 62, 63, 64, 67, 68, 69, 70, 73, 74, 83, 87
V	5	Expert opinion	1	76
		Review	6	8, 9, 16, 31, 37, 84
0	0	Animal study	10	12, 14, 15, 17, 23, 33, 71, 82, 98, 100
		Technical note	2	61, 80

List Comparisons

The list of 100 most relevant articles shows several differences compared with the list of 100 most cited articles. A significant number (n=41) of articles on the most relevant list are not included in the most cited list. Of the 59 articles remaining on both lists, 41 are among the top 50 on the most cited list.

The most relevant list, not surprisingly, contains considerably more recently published articles than the most cited list. As shown in Figure 1, there is a lag of almost a decade before the steep increase in the number of the most relevant articles compared to that of the most cited articles. Though both plots peak in the 1990s, the one representing the most relevant list has a steadier decrease thereafter. The median year of publication is 1999 for the 100 most relevant articles, compared to 1990 for the 100 most cited articles (P < 0.0001). In addition, the most relevant list shows a more equitable pattern of article topic distribution than the most cited list (Table 3). While the most represented topic remains cerebrovascular (n=36), there is a significant increase in the number of articles reporting tumor studies (30 vs. 18). Compared with the most cited list wherein five out of seven papers addressing glioma were related to malignant glioma, the most relevant list contains more studies on low-grade glioma, specifically with three articles addressing awake surgery with cortical and subcortical electrical mapping originating from the same team in France (numbers 34, 68, and 74 in Table 2). Additionally, there are two articles concerning radiosurgery for vestibular schwannoma and melanoma brain metastases, respectively (numbers 49 and 88 in Table 2). Though the most relevant list has a similar number of articles on functional neurosurgery compared with the most cited list (12 vs. 11), it has a broader coverage of disorders (e.g. epilepsy) and procedures (e.g. vagus nerve stimulation). There are fewer trauma articles in the most relevant list (20 vs. 26).

With regard to the type of article and the associated level of evidence, the most relevant list consists of twice as many review articles as the most cited list (14 vs. 7) (Table 3). Of note, six articles are systematic reviews/meta-analyses which correspond to level I/1 (NHMRC/CEBM) evidence. The number of prospective studies (n=32) increased in the most relevant list while the number of retrospective studies (n=37) decreased, corresponding to level III/2 and level IV/4 (NHMRC/ CEBM) evidence, respectively. The most relevant list has a similar number of randomized controlled trials (n=6) and literature reviews (n=8) compared with the most cited list which corresponds to level II/1b and V/5 (NHMRC/CEBM) evidence, respectively.

There seems to be no dramatic discrepancy regarding the country of origin of articles between the two lists (Table 3). With regard to institutional contributions, University of Pittsburgh Medical Center led the list with nine articles covering all four major topics, followed by the University of California at San Francisco with eight articles (Figure 4). Authors who contributed three or more articles to the most relevant list are shown in Table 7 with their position on the author list.

**Table 7:** Authors contributing three or more articles to the 100 most relevant articles * Refers to the article number in Table 2 † Rank last in the author list as corresponding author

Name	No. of articles	Position on author list	Article Number*
Berger, Mitchel S	6	Fourth (1), Seventh (2), Eleventh (1), Senior† (2)	2, 17, 28, 41, 57, 97
Vinuela, Fernando	5	First (1), Second (3), Eighth (1)	8, 16, 18, 31, 94
Duckwiler, Gary	4	Second (1), Third (2), Fourth (1)	8, 16, 18, 94
Guglielmi, Guido	3	First (3)	18, 31, 94
Marmarou, Anthony	3	First (1), Second (1), Sixth (1)	9, 36, 38
Becker, Donald P	3	Second (1), Sixth (1), Eleventh (1)	27, 38, 99
Young, Harold F	3	Fifth (1), Seventh (1), Eighth (1)	36, 38, 86

## Discussion

As the official journal of AANS and one of the top journals in the field of neurosurgery, JNS has published over 21,933 articles since 1944. Its significant academic influence and considerable contribution to the evolution of modern neurosurgery has been recognized not only by the steadily increasing JCR impact factor scores, but also by previous citation analyses on the top cited and most relevant works in neurosurgery. These analyses identified JNS as the number one neurosurgical journal on both lists, contributing 79 articles to the 100 top cited list and 63 articles to the 100-most relevant list among all neurosurgical journals [3-5]. However, analysis remains elusive regarding the articles, authors, institutions, and topics that are driving the achievement and progress of JNS.

This bibliometric analysis identified the 100 most cited and most relevant articles published in JNS since its first issue launched in 1944. The two lists represented here may serve as the most objective and reliable resources in defining the classic and foundational works of the journal as well as the field of neurosurgery to some extent, because this modality is less influenced by subjective bias [14]. Indeed, a set of seminal articles which have had significant influence over both practice and research in the field of neurosurgery were included in the lists. These may also serve as fundamental reading lists for the education of neurosurgical residents.

Both the 100 most cited and most relevant lists displayed a so-called “90s peak” regarding the temporal trends in published articles, which has been reported in previous citation analyses as well [4, 15]. This phenomenon may be explained by several factors including (1) the transition point where older works are losing their relevance in modern practice to newer works just beginning to accumulate citations; (2) a dramatic increase in citation indexing boosted by online databases which emerged around 2005 [4]; and (3) an expanding number of neurosurgeons and researchers in the field which may result in increased citations of more recently published articles. In addition, there seems to be a predictable course for articles to accumulate citations around two years after publication, peak after three to ten years, and then decline over time [4, 16]. It would then be expected that articles published in the most recent decade will accumulate citations and peak in the following years. The strategy to identify the 100 most relevant articles by ranking the literature according to citations per year has to some extent overcome the inherent problem of citation analyses which favors older publications by using total citations as a measure of impact [5, 17]. 

It is not surprising that institutions from North America contributed to the majority of articles on both lists. The disparity in numbers between the US and other countries may be attributed to several reasons including (1) the inherent association of AANS with JNS as official journal; (2) the fact that government funding and resources allocation for research was several times higher in the US than other countries [18-19]; (3) the possible bias associated with our classification strategy according to the corresponding author’s institution as many studies enjoy multi-center cooperation; and (4) the inherent bias of the JNS being an English-language publication and the US as a primarily English-speaking nation (at least in matters of scientific discovery). However, international institutions located in 131 countries from other continents have made considerable contributions as well, which further validate the academic influence of JNS worldwide. This analysis also highlighted the significant contribution of many neurosurgeons to the growth and achievements of JNS. The contributors listed herein should not only be considered as a memorial for young generations to admire, but also as an impetus to inspire.

This bibliometric analysis also revealed the characteristics and trends of research in neurosurgery, which may also shed light on the direction for future research. The 100 most cited and most relevant lists both showed a strong focus on cerebrovascular disorders and procedures, with the latter group featuring more large-scale and well-designed studies on new techniques or first-hand experiences with new devices, such as Onyx injection for intracranial AVM, flow diverters, stents, indocyanine green videoangiography for aneurysm surgeries, and neurotransplantation for stroke patients. Similarly, distinguishing characteristics of the two lists concerning the topic of tumor are revealed by the significant increase in the number of articles related to the newly developed technique of awake surgery with functional electrical mapping for patients with low-grade glioma, the advancement of endoscopic endonasal approaches, surgical techniques for resection of a wider range of skull base tumors, and the application of radiosurgery for treatment of a variety of benign and malignant brain tumors.

With regard to functional neurosurgery, which represents one of the fastest-developing subspecialties, articles on management of refractory mood disorders and seizure disorders with stimulators emerged in the most relevant list compared with the most cited list, while trauma studies, which are thought to lose some of their popularity to more current hot topic research areas, did decrease in total numbers from 26 in the most cited list to 20 in the most relevant list. Of note, there were more articles on mild TBI and laboratory research on pathophysiology as well as cutting-edge diagnostic and therapeutic (in animal models) modalities in the latter group.

Though the majority of articles were original clinical studies, there were seven and 14 review articles among the 100 most cited and most relevant articles, respectively. Six systematic reviews/meta-analyses, which were exclusively included in the most relevant list, corresponded to level I evidence and could influence clinical practice and research directions as they are able to pool data from individual under-powered studies with disagreeing results and draw conclusions using statistical tools. Alternatively, the importance of literature review articles was noted in both lists as they offer a current overview of the field with synthesis of related individual studies and/or expert opinions on a specific topic [4]. 

The majority of articles in the 100 most cited list corresponded to level III (original prospective studies, n=28) and level IV (retrospective studies, n=46) evidence. Of note, there was no systemic review/meta-analysis representing level I evidence, and there was only one article in the top 20 with high-level evidence (I or II) as a randomized controlled trial, which seemed fewer in number than previous citation analyses on other subspecialty journals [6, 20-21]. However, the overall number of articles with high-level evidence increased significantly in the most relevant list, with six systemic reviews/meta-analyses and six randomized controlled trials, which was higher than other subspecialized analyses [6, 22] and reflected the trend of increasing high-quality studies valued by the scientific community. 

With the fact of more recently published articles and more level I evidence included in the most relevant vs. the most cited list, it is important to acknowledge unique characteristics and the usefulness of each list. The most cited list provides a better blueprint of the foundation and development of the field from a more historical perspective, while the most relevant list points to the science underlying current practice and future directions [3]. Either of the two lists can be used under different circumstances. With the push for evidence-based medicine that started in the 1990s, lists such as these will be very important in substantiating current practice methods, modification of practice, surgical techniques, and quality of care. If viewed from within this framework of evidence-based practice, bibliometric studies such as this one will become the tools that demonstrate neurosurgery's commitment to evidence-based practice to the world. It can also be easily inferred that with data provided from bibliometrics analysis such as these, the medical establishment can also exert the necessary influence in things such as insurance coverage of treatments deemed "experimental", reimbursements, and encourage a decrease in unnecessary procedures.

There are several limitations to this bibliometric analysis. First, some important recent publications with high numbers of citations per year but relatively low total citations may have escaped detection by our search strategy. Alternatively, the relevance of these articles may need to be tested by time according to the pattern of accumulating citations. Furthermore, with the evolution of a single JNS into the JNS publishing group, the official journals of AANS now include an additional three independent journals. This includes (1) Journal of Neurosurgery: Spine (since 2004) focusing on spinal surgery; (2) Journal of Neurosurgery: Pediatrics (since 2008) focusing on neurological diseases and disorders in children, and (3) Neurosurgical Focus (since 1996) covering a specific neurosurgical topic in depth for each issue. The current analysis limited its search to JNS only, and thus may have missed articles related to spine surgery, pediatric neurosurgery, and reviews published in the other three series. Additionally, certain landmark works may not have been captured if they have appeared in a textbook or published in a language other than English. Last but not least, as a “snapshot” analysis of published literature and their academic impact, any list created by citation analysis is dynamic and may change due to the function of time.

## Conclusions

This bibliometric analysis identified the 100 most cited articles originating in JNS by total citation counts and the 100 most relevant articles in JNS by citations per year. The findings revealed the key contributing factors to the growth and flourish of JNS as well as classic and foundational works in the field of modern neurosurgery. Discrepancies between the most-cited and most-relevant lists also suggested that studies incorporating newly developed modalities, high-quality clinical trials and laboratory investigations, and systematic reviews with level I evidence are of growing interest and will likely be areas of continued growth in the future. Bibliometric analysis serves as a useful tool to establish benchmarks in the development of the field and to propose career directions in future research and practice.
